# Supplementary material for: The interactome of intact mitochondria by cross-linking mass spectrometry provides evidence for coexisting respiratory supercomplexes
Source: Mol Cell Proteomics. 2017 Dec 8;17(2):216–32. doi: 10.1074/mcp.RA117.000470 (PMC5795388; doi:10.1074/mcp.RA117.000470)
Supplement: Supplemental Data [file supp_RA117.000470_133922_0_supp_23978_fzffwf.zip › spectra_annotation/mito_spectra_annotation/446-1-6-1-2-1.pdf]

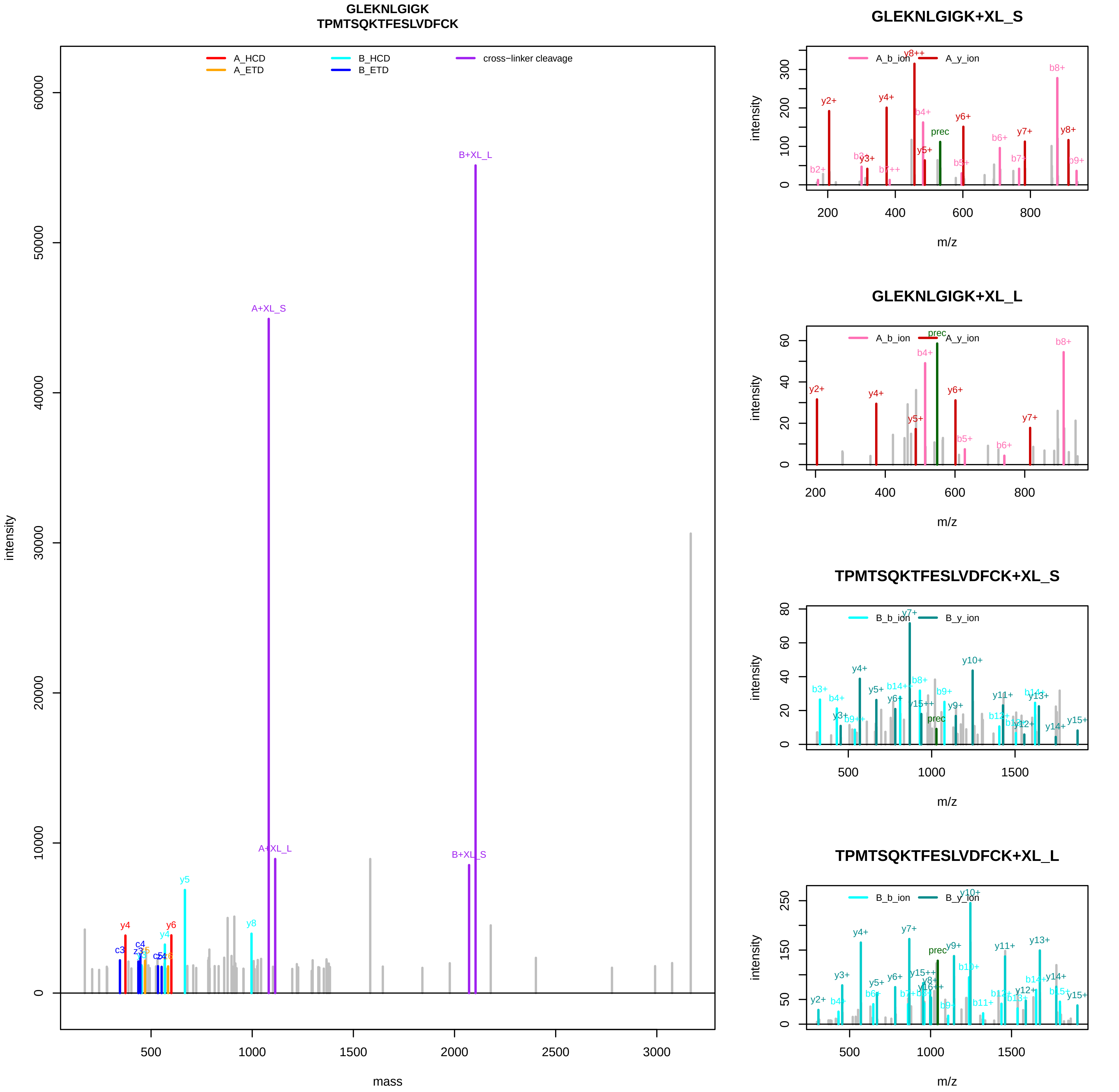
GLEKNLGIGK
TPMTSQKTFESLVDFCK
A_HCD
A_ETD
B_HCD
B_ETD
cross−linker cleavage
B+XL_L
A+XL_S
A+XL_L
B+XL_S
y4
y6
y5
y8
intensity
mass
0
10000
20000
30000
40000
50000
60000
500
1000
1500
2000
2500
3000
GLEKNLGIGK+XL_S
A_b_ion
A_y_ion
y2+
y4+
y8++
b4+
prec
y6+
b6+
y7+
y8+
b8+
b9+
b2+
b3+
y3+
y5+
b5+
b7+
m/z
GLEKNLGIGK+XL_L
prec
b4+
b8+
y2+
y4+
y5+
y6+
b5+
b6+
y7+
TPMTSQKTFESLVDFCK+XL_S
B_b_ion
B_y_ion
y7+
y4+
y10+
b3+
b4+
y5+
b8+
b9+
y9+
y11+
y13+
prec
y14+
y15+
TPMTSQKTFESLVDFCK+XL_L
y10+
y4+
y7+
y13+
prec
y9+
y11+
y3+
y14+
y2+
y15+
